# Low levels of IgM antibodies to oxidized cardiolipin increase and high levels decrease risk of cardiovascular disease among 60-year olds: a prospective study

**DOI:** 10.1186/1471-2261-13-1

**Published:** 2013-01-07

**Authors:** Jun Su, Xiang Hua, Max Vikström, Karin Leander, Bruna Gigante, Mai-Lis Hellenius, Ulf de Faire, Johan Frostegård

**Affiliations:** 1 Institute of Environmental Medicine, Unit of Immunology and Chronic Disease, Karolinska Institutet, 17177 Stockholm, Stockholm, Sweden; 2 Division of Cardiovascular Epidemiology, Institute of Environmental Medicine, Karolinska Institutet, Stockholm, Sweden; 3 Department of Medicine, Karolinska University Hospital, Solna, Sweden; 4 Department of Cardiology, Karolinska University Hospital, Solna, Sweden

**Keywords:** Cardiovascular disease, Cardiolipin, Oxidation, Antibodies

## Abstract

**Background:**

Antibodies against cardiolipin (aCL) are associated with increased risk of cardiovascular disease (CVD). We here determine the role of antibodies against oxidized CL (aOxCL).

**Methods:**

One third of sixty-year olds from the Stockholm County were screened (2039 men, 2193 women), where 211 incident CVD-cases and 633 age- and sex-matched controls were identified (5–7 year follow-up). Antibodies were determined by ELISA and uptake of oxLDL in macrophages by FACScan.

**Results:**

IgM aOxCL was lower among CVD cases than controls (p=0.024). aOxCL-levels were divided in quartiles with the highest quartile set as the reference group. After adjustment for smoking, BMI, type II diabetes, hypercholesterolaemia and hypertension, an increased risk was determined in the lowest quartile of IgM aOxCL (OR: 1.80, CI: 1.12–2.91, p=0.0159); OR for men in the lowest quartile was 2.46 (CI 1.34–4.53, p=0.0037) for CVD and for stroke: 12.28 (CI: 1.48-101.77, p=0.02). IgG aOxCL levels did not differ between quartiles in CVD-risk. High levels of IgM aOxCL (reaching significance above 86th) and IgG aOxCL (above 95th percentile) were associated with decreased risk of CVD (OR: 0.485, CI: 0.283-0.829; p=0.0082 and OR: 0.23, CI: 0.07-0.69; p=0.0091). aCL were not associated with CVD. oxCL but not CL competed out uptake of OxLDL in macrophages, and aOxLDL recognized oxCL but not CL. In contrast to aCL, aOxCL was not dependent on co-factor Beta2-glycoprotein-I.

**Conclusions:**

aOxCL is a novel risk/protection marker for CVD, with therapeutic implications. OxCL competes with oxLDL for uptake in macrophages and the possibility that aOxCL inhibits such uptake by interfering with same or similar epitopes in oxCL and oxLDL should be further studied.

## Background

Atherosclerosis is the major underlying cause of cardiovascular disease (CVD) as stroke and myocardial infarction (MI) and can be regarded as an inflammatory disease, where activated immune competent cells producing cytokines are typical features [1]. However, traditional risk factors as age, male sex, hypertension, hyperlipidemia, smoking and diabetes do not account for the inflammatory nature of atherosclerosis. Therefore, novel risk markers are needed which account for inflammation and immune reactions related to atherosclerosis and CVD. High sensitivity C-reactive protein (hsCRP) has been much discussed and is of major interest in CVD [2]. However, the volatility of this measure may be a limitation when hsCRP is used at the individual level. LDL-PLA2 is another interesting emerging inflammatory risk marker [3]. We have recently reported that natural antibodies against phosphorylcholine of IgM subclass (anti-PC) could be of interest, since low levels of these antibodies in several studies were associated with increased risk of CVD [4-8]. Since current therapies in atherosclerosis and CVD were not developed to target the inflammatory and immunological nature of these conditions, also therapies with anti-inflammatory and/or immune modulatory properties are needed.

CL is a phopholipid with a unique double phospholipid, containing four fatty acid chains. CL is found primarily in the inner mitochondrial membrane of euraryotic cells and in bacteria [9] which is interesting to note since mitochondria are believed to have a bacterial origin from an evolutionary point of view [10]. CL plays a central role in mitochondrial bioenergetics and also appears to be of major importance in apoptosis and membrane dynamics [9].

Antibodies against CL (anti-CL) are generally recognized as risk factors for thrombosis, both venous and arterial, especially in patients with rheumatic diseases like systemic lupus erythematosus (SLE) [11].

To the best of our knowledge, the clinical role of antibodies against oxidized CL (OxCL) has not been described previously. We here report that anti-OxCL in contrast to anti-CL is negatively associated with CVD: low levels being associated with increased risk and high levels with decreased risk. The implications of these findings are discussed.

## Methods

### Subjects

From July 1^st^ 1997 to June 30^th^ 1998, every third man and woman living in the County of Stockholm reaching the age of 60 years, were invited to participate in a health screening for cardiovascular diseases. By this selection of individuals, age bias was avoided. A total number of 4232 subjects (2039 men and 2193 women; response and rate 78%) participated in the investigation. Information on sociodemography, lifestyle habits, medication and previous diseases and hospitalizations was obtained by a self-administered questionnaire. Physical examination with blood pressure measurements, anthropometry and ECG was performed and serum, plasma and whole blood were collected for storage in a biological bank (−80°C). Details of the screening procedure have been given elsewhere [12,13]. The study was approved by the Karolinska Institutet research ethics committee and is in accordance with the Helsinki Declaration. All subjects gave written informed consent before entering the study.

### A nested case–control design

To record incident cases of first CVD, new events of coronary heart disease (CHD), defined as fatal and non-fatal myocardial infarction (MI) and ischemic stroke, hospitalization for angina pectoris, were registered. The study base of 4232 subjects was matched with the national cause of death registry (fatal events until December 31, 2003) and the national in-hospital registry (non-fatal events until December 31, 2005 ). Through these matching procedures 211 incident cases of CVD were recorded. To guarantee that first CVD events were registered, only subjects without a history of CVD prior to recruitment were utilized for the matching procedures. The International Classification of Diseases (ICD-10) was used to register CHD-deaths (I 20, I 21, I 46), MI (I 21), angina pectoris including PCIs and CABGs (I 20, Z 95.5 and Z 95.1) and ischemic stroke (I 63-I 66). The criteria used were thus uniform and consistent. For each case three controls were randomly selected, matched for gender and age (+/− 60 days). Thus, a nested case–control design (211 cases and 633 controls) was applied for the epidemiological and statistical analyses.

### Oxidation of CL

CL from bovine heart was purchased as ethanol solution from Sigma (Sigma product C 1649) and was stored at −20°C. To generate saturated molecular species, cardiolipin was oxidized in aqueous solutions containing 1.5 mmol/L tert-butylhydroperoxide and CuSO_4_ in 20 μmol/L. Both CL and thus treated CL were measured with mass spectrometry (Electrospray ionization mass spectrometer (Micromass, Beverly, MA, USA), to confirm that CL had been oxidized by copper and tert-butylhydroperoxide.

### Determination of antibodies against CL and OxCL with ELISA

IgM and IgG antibodies to OxCL were determined by enzyme-linked immunosorbent assay (ELISA). serum from two donors with anti-OxCL levels above median levels were used as internal standard and tested on every plate. The plateau of antibody binding was reached with the antigen concentration of 10 μg/ml. Immulon 1B plates (Thermo Labsystems, Franklin, MA, USA) were coated with oxidized cardiolipin (OxCL) (10 μg/ml) 50 μl/well in ethanol. Coated plates were incubated overnight at 4°C. After five washings with PBS, the plates were blocked with 2% BSA-PBS for 2h at room temperature and washed as described above. Serum samples were diluted (1:50) in 0.2% BSA-PBS and added at 50 μl/well.

Plates were incubated overnight at 4°C and washed as described above. Alkaline phosphatase conjugated goat anti-human IgM or IgG (diluted 1:7000 in the sample buffer) were added at 100 μl/well and incubated at 4°C overnight. After five washings, color was developed by adding the alkaline phosphatase substrate (PNPP) at 100μl/well and incubating the plates for 60 min at room temperature in the dark. The plates were read in an ELISA Multiscan Plus spectrophotometer at 405 nm. All samples were measured in duplicates a single assay and the coefficient of variation was below 10%.

IgM and IgG antibodies to CL where measured by a standard ELISA kit (Orgentec diagnostika GmbH, Mainz, Germany) according to the manufacturers description.

In order to investigate the specificity of anti-OxCL, competition assays were performed. At a dilution giving 50% of maximal binding to anti-OxCL, sera were preincubated with different concentrations of OxCL or CL coated overnight onto glass tubes. After vortexing, the tubes were incubated at 4°C overnight and centrifuged at 13000 r.p.m. for 30 min (4°C). The supernatants were tested for antibody binding to OxCL as described.

To test effects of aOxLDL, sera were pre-incubated with different concentrations of OxLDL (Intracel, Frederick, Maryland) at 4°C overnight and centrifuged at 13000 r.p.m. for 30 min (4°C). The supernatants were tested for antibody binding to OxLDL, OxCL and CL.

The percentage of inhibition was calculated as follows:

Percent inhibition = (OD without competitor – OD with competitor)/OD without competitor × 100

### Effect of ß2GPI on the binding of anti-OxCL or anti-CL with ELISA

The assay was essentially performed as described above for the anti-OxCL or anti-CL ELISA. Plates were coated with OxCL or CL, blocked with 2% BSA-PBS, then incubated with different concentration of ß2GPI or diluent alone with sera from high-titer patients from patients with the antiphospholipid antibody syndrome (APS) for 1h at room temperature. Each value shown is the mean±standard deviation.

### Inhibition of macrophage uptake of oxLDL

oxLDL prepared by incubation with CuSO4 (Intracel, Frederick, US) was incubated with Dil (Molecular Probes Engene, Oregon, USA) in lipoprotein-deficient serum (Sigma) at 37°C for 15 hours. Then the mixture was dialyzed against saline-EDTA buffer for 6 hours.

The macrophages differentiated from THP-1 cells were plated in a 24-well plate (NUNC Inc, Naperville, Ill) at density of 3 × 10^5^ cells/well in DMEM (INVITROGEN, USA) containing 10% FBS overnight. Then the cells were incubated with Dil-oxLDL (5 μg/ml), with oxCL (7, 10 μg/ml), with CL control (7, 10 μg/ml) for 4 hours. Thereafter, the cells from above were washed 4 times with 0.2% BSA/PBS and once with PBS. The cells were harvested in PBS containing 0.1% BSA and 0.01% NaN3. Uptake of Dil-labelled oxLDL was studied by Flow cytometry and a minimum of 10.000 cells was analyzed.

### Determination of estimated glomerular filtration (eGFR) and risk score

As a measurement of GFR, we used the estimated GFR (eGFR) using the CKD-EPI formula [14]. For determination of risk score, we used the Framingham risk score (formula) [15].

### Statistics

Demographic data, biochemistry and anthropometry were performed for cases and controls respectively with values expressed as mean ± standard deviations (SD) for normally distributed parameters and medians (ranges) or proportions for parameters which were not normally distributed after logarithmic transformation. Statistical differences between cases and controls were assessed through non-parametric tests. Odds ratios (OR) with 95% confidence intervals (CI ) were calculated applying conditional logistic regression. Analyses were run crude or adjusted for traditional risk factors as indicated. Receiver operating characteristic (ROC) curves and the area under those curves (AUC) for stroke prediction with and without anti-PC were calculated.

Statistical analyses were run with SAS®statistical software system version 9.1.

## Results

**Figure 1:**
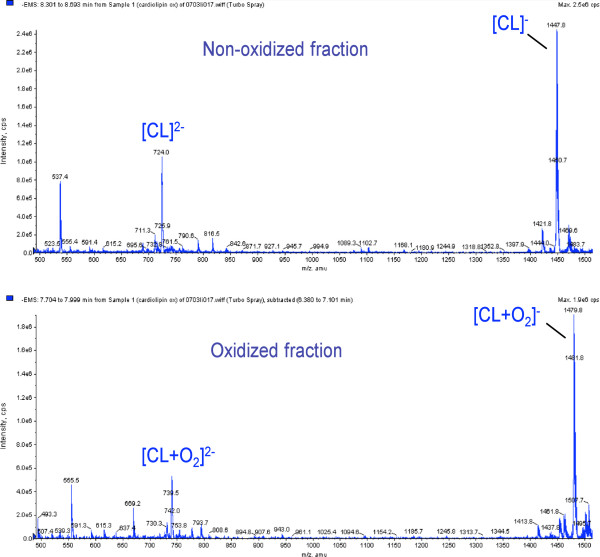
**Electrospray ionization mass MS spectrometer **(**Micromass**, **Beverly**, **MA**) **was used to demonstrate that cardiolipin was oxidized.**

**Figure 2:**
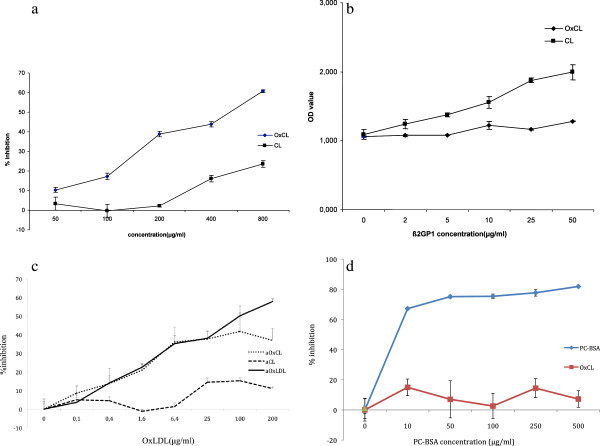
**a Competition ELISA of IgM anti**-**OxCL binding to plated OxCL in the absence or presence of indicated amounts of OxCL or CL preincubated with sera. **The results are expressed as Percent inhibition = (OD without competitor - OD with competitor)/ OD without competitor X100. Each value shown is the mean±standard deviation. **b** ELISA of IgM anti-OxCL or anti-CL which had been postincubated with different concentration of ß2GP1 (for 1 hour at RT). ß2GPI could increase anti-CL binding to CL but not anti-OxCL binding to OxCL. The results are expressed as mean±standard deviation. **c** Competition ELISA of binding of IgM anti-OxCL, anti-CL and aOxLDL respective antigens after preincubation with the indicated concentrations of oxLDL. The results are expressed as Percent inhibition = (OD without competitor - OD with competitor)/ OD without competitor X100. Each value shown is the mean±standard deviation. **d** Competition ELISA of binding of IgM anti-OxCL and anti-PC to respective antigens after preincubation with the indicated concentrations of PC-BSA. The results are expressed as Percent inhibition = (OD without competitor - OD with competitor)/ OD without competitor X100. Each value shown is the mean±standard deviation.

CL-oxidation by copper and tert-butylhydroperoxide was confirmed by mass spectrometry (Figure 1). Preincubation with OxCL could inhibit up to 60-70% of IgM anti-OxCL binding to OxCL while CL (exposed to air over night had low capacity to influence anti-OxCL binding (Figure 2a). Further, ß2GPI, a co-factor for anti-CL, could induce increased anti-CL binding to CL but not anti-OxCL binding to OxCL (Figure 2b). 

OxLDL competed out IgM antibody binding to oxCL but effects on binding to CL were negligible (Figure 2c). PC-BSA competed out IgM antibody binding to PC-BSA but not to oxCL (Figure 2d).

We identified 211 incident cases of first CVD events throughout the follow-up period (77 with MI, 85 with angina pectoris and 49 with ischemic stroke). For each incident case three age and sex matched controls were selected, and thus 633 controls. Serum samples were missing for 2 case and 18 controls leaving 209 cases and 615 controls for analyses.

As previously reported, there were more smokers and hypertensives among cases than controls and trendwise higher BMI. Further, blood pressure levels, blood lipids, hCRP and apoproteins were less favourable among cases as compared to controls (Table 1). 

**Table 1:** Baseline characteristics among incident CVD cases and matched controls

	**Incident cases**	**Controls**	**P value**
Number	211	633	NA
Age, years	60	60	NA
Male gender, %	66.4	66.4	NA
Smokers, %	31.9	19.6	0.0002
Diabetes %	24.3	15.7	0.005
BMI kg/m2	27.7 ±4.6	26.7 ±3.8	0.0031
Hypertension (>140/90 mm Hg ), %	42.4	25.6	<0.0001
Glucose mmol/L	6.1 ±2.5	5.6 ±1.5	0.0004
Insulin μmol/L	11.4 ±7.1	10.2±6.5	0.0317
Systolic blood pressure, mm Hg	148 ±21.8	139 ±21.2	<0.0001
Diastolic blood pressure, mm Hg	98 ±10.6	85 ± 10.4	<0.0001
Cholesterol, mMol/l	6.1 ±1.0	6.0 ±1.2	0.14
HDL, mMol/l	1.3 ±0.4	1.4 ±0.4	0.0005
LDL, mMol/l	3.9 ±1.2	3.8 ±1.1	0.45
Triglycerides, mMol/l	1.6 ±1.0	1.4 ±0.8	0.0005
hsCRP, mg/l	2.4 (1.3-4.7)	1.7(0.9-3.2)	<.0001
eGFR	90.5 (81.4-106.0)	90.9 (80.4-103.9)	0.73
Framingham Risk Score	14.5±2.3	13.5±2.0	<0.0001
IgM anti-OxCL U/ml, men and women.	88.97(68.36-114.6)	96.90(72.56-123.44)	0.024
IgM anti-OxCL U/ml , men.	84.16(63.38-112.57)	96.90(70.35-123.10)	0.0047
IgM anti-OxCL U/ml , women.	99.06(78.04-123.70)	96.75(74.07-124.37)	0.78
IgM anti-CL U/ml, men and women.	1.87(1.34-3.00)	2.02(1.38-3.12)	0.34

Data are presented as percentage, mean ± SD, or median with interquartile ranges within paranthesis.

Median values and interquartile ranges for IgM anti-OxCL indicated lower values among incident cases than controls, a difference particularly apparent in men where incident cases had significantly lower median values as compared to controls, (Table 1).

The association between anti-OxCL and risk of CVD indicates an excess risk for those within the lowest quartile of anti-OxCL values compared with subjects within quartile 4 (set as the reference group, OR = 1.0). Even though OR in quartile 2 and 3 were also raised, this difference did not reach statistical significance. Also after adjustment for smoking, BMI, type II diabetes, hypercholesterolaemia, and high blood pressure a significantly increased relative risk (OR) for CVD was determined in the lowest quartile of anti-OxCL (Tables 2, 3, and 4). 

**Table 2:** **Association between levels of IgM anti**-**OxCL and risk for MI and**/**or **+ **stroke **(**CVD**), **men and women**

**anti**-**OxCL**					**Crude**				**Adjust**			
Quartiles	(U/ml)		cases	controls	OR	95% CI		P-values	OR	95% CI		P-values
Quartile 4	122.16>		41	165	1	N/A		NA	1	N /A		N /A
Quartile 3	94.99<	<=122.16	55	151	1.47	0.93	2.33	0.099	1.58	0.98	2.55	0.059
Quartile 2	70.65 <=94.99		52	154	1.41	0.88	2.26	0.16	1.43	0.88	2.33	0.15
Quartile 1	<=70.65		61	145	1.75	1.10	2.79	0.018	1.80	1.12	2.91	0.016

**Table 3:** **Association between levels of IgM anti**-**OxCL and MI and**/**or **+ **stroke **(**CVD**), **men**

**anti**-**OxCL**					**Crude**				**Adjust**			
Quartiles	(U/ml)		cases	controls	OR	95% CI		P-values	OR	95% CI		P-values
Quartile 4	122.16>		23	107	1	NA		NA	1	NA		NA
Quartile 3	94.99<	<=122.16	35	107	1.52	0.85	2.73	0.16	1.66	0.90	3.06	0.11
Quartile 2	70.65	<=94.99	33	97	1.65	0.89	3.06	0.11	1.80	0.94	3.42	0.075
Quartile 1	<=70.65		48	104	2.22	1.24	3.98	0.0070	2.46	1.34	4.53	0.0037

**Table 4:** **Association between levels of IgM anti**-**OxCL and MI and**/**or **+ **stroke **(**CVD**), **women**

**anti**-**OxCL**					**Crude**				**Adjust**			
Quartiles (U/ml)			cases	controls	OR	95% CI		P-values	OR	95% CI		P-values
Quartile 4	122.16>		18	58	1	N/A		N /A	1	NA		NA
Quartile 3	94.99<	<=122.16	20	44	1.49	0.70	3.20	0.30	1.67	0.76	3.67	0.20
Quartile 2	70.65	<=94.99	19	57	1.14	0.54	2.42	0.72	1.14	0.52	2.47	0.75
Quartile 1	<=70.65		13	41	1.04	0.45	2.40	0.92	0.99	0.42	2.32	0.97

*The OR and the P-values for each quartile are compared to quartile 4.

If the highest 25th percentile of IgM aOxCL were compared with the other three lower percentiles, the risk of the lower three was raised significantly for the whole group (crude: OR 1.54, CI 1.04-2.28, p=0.031; adjusted OR 1.60, CI 1.07-2.40, p= 0.022). These associations also reached significance for men (crude: OR 1.77, CI 1.07-2.57, p=0.025; adjusted OR 1.94, CI 1.15-3.30, p= 0.014) but not for women (data not shown).

If the lowest 25th percentile of IgM aOxCL were compared with the other three higher percentiles, the risk of the lower was raised trendwise for the whole group (crude: OR 1.36, CI 0.95-1.95, p=0.090; adjusted OR 1.37, CI 0.94-1.98, p= 0.10). These associations reached significance for men (crude: OR 1.59, CI 1.05-2.43, p=0.030; adjusted OR 1.606, CI 1.07-2.57, p= 0.024) but not for women (data not shown).

Women in the highest quartile were significantly protected against MI (data not shown).

High levels of IgM anti-OxCL, adjusted for smoking, BMI, type II diabetes, hypercholesterolaemia, and high blood pressure (reaching significance above 86th percentile) were associated with significantly decreased risk (OR: 0.485, CI 0.0.283-0.829; p=0.0082).

Further adjustements for hsCRP gave essentially similar OR for IgM anti-OxCL (data not shown). Regarding co-morbidities such as autoimmune diseases, there were 10 individuals with Rheumatoid Arthritis. These did not differ significantly from others in antibody levels (data not shown).

There were no significant associations between IgM anti-CL and CVD in our study (data not shown). However, trendwise, very high anti-CL levels were associated with increased risk of CVD among men, and above highest 98^th^ percentile OR was 2.37 for women, (non-significant).

There were no significant differences between cases and control in levels of anti-OxCL or anti-CL, when median levels where compared (data not shown).

High levels of IgG anti-OxCL (above 95th percentile), adjusted for smoking, BMI, type II diabetes, hypercholesterolaemia, and high blood pressure were associated with strikingly and significantly decreased risk (OR=0.23: CI 0.07-0.69; p=0.0091).

There were no significant associations between IgG anti-CL and CVD in our study (Tables 5, 6 and 7). Further, very high IgG anti-CL levels, above 98th percentile (as in the antiphospholipid antibody syndome) were not associated with increased risk of CVD (OR=0.94, CI=0.29-3.09 P=0.9206). 

**Table 5:** **Association between levels of IgG anti**-**OxCL and risk for MI and**/**or **+ **stroke **(**CVD**), **men and women**

**aOxC**.**IGG**					**Crude**			**Adjust**		
Quartiles	(U/ml)		cases	controls	OR	95% CI	P-values	OR	95% CI	P-values
Quartile 4	80.81>		53	155	1	N/A	NA	1	N /A	N /A
Quartile 3	59.66<	<=80.81	49	156	0.89	0.57-1.39	0.62	0.95	060-1.51	0.83
Quartile 2	44.13<	<=59.66	55	153	1.05	0.67-1.63	0.84	1.26	0.79-2.02	0.33
Quartile 1	44.13<=		52	151	0.99	0.63-1.54	0.95	1.11	0.70-1.77	0.65

**Table 6:** **Association between levels of IgG anti**-**OxCL and MI and**/**or **+ **stroke **(**CVD**), **men**

**anti**-**OxCL**.**IGG**					**Crude**			**Adjust**		
Quartiles	(U/ml)		cases	controls	OR	95% CI	P-values	OR	95% CI	P-values
Quartile 4	80.81>		38	111	1	NA	NA	1	NA	NA
Quartile 3	59.66<	<=80.81	34	111	0.89	0.53-1.50	0.66	0.98	0.56-1.69	0.93
Quartile 2	44.13 <	<=59.66	41	100	1.21	0.72-2.03	0.47	1.45	0.84-2.51	0.18
Quartile 1	44.13<=		26	93	0.83	0.47-1.45	0.51	0.94	0.53-1.68	0.83

**Table 7:** **Association between levels of IgG anti**-**OxCL and MI and**/**or **+ **stroke **(**CVD**), **women**

**anti**-**OxCL**.**IGG**					**Crude**			**Adjust**		
Quartiles	(U/ml)		cases	controls	OR	95% CI	P-values	OR	95% CI	P-values
Quartile 4	80.81>		15	44	1	N/A	N /A	1	NA	NA
Quartile 3	59.66<	<=80.81	14	45	0.90	0.38-2.11	0.81	0.92	0.37-2.19	0.82
Quartile 2	44.13<	<=59.66	14	53	0.76	0.32-1.82	0.54	0.96	0.39-2.38	0.93
Quartile 1	44.13<=		26	58	1.22	0.55-2.69	0.63	1.39	0.60-3-20	0.44

*The OR and the P-values for each quartile are compared to quartile 4.

The AUC of ROC curves accounting for traditional risk factors (hypertension, diabetes, hypercholesterolemia, smoking and BMI) was 0.63 for the whole study group, 0.63 for men and 0.65 for women. Adding IgM anti-OxCL (with cut-off below 25th percentile) to this model gave AUC of 0.64 for the whole group, 0.64 for men and 0.66 for women. the increase among men was significant in the maximum likelihood ratio test (p=0.02).

**Figure 3:**
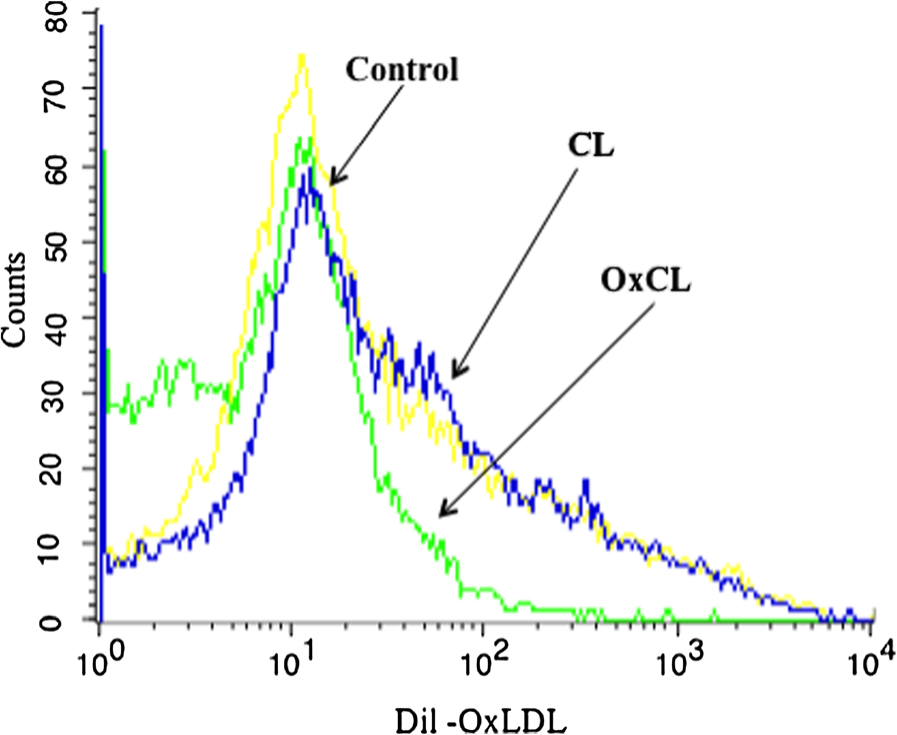
**Effect of OxCL on macrophage uptake of OxLDL. **The histograms depict differences in fluorescence intensity of Dil-OxLDL taken up by macrophages with 5ug/ml of CL or OxCL, indicating that OxCL but not CL decreases the uptake of OxLDL. Yellow represents control with no addition of lipid or other compound.

To study further potential mechanisms of oxCL bioactivity, 1×10^6^/ well THP-1 differentiated macrophages were used for uptake studies. oxLDL could inhibit dil-OxLDL uptake up to more than 60%, but LDL hade almost no effect on the uptake of dil-oxLDL which confirmed the specificity of uptake results. Further results indicate that oxCL can, in a concentration dependent manner, inhibit dil-oxLDL uptake while the native CL did not show the competition effects (Figure 3). 

## Discussion

The main finding in this study is that IgM antibodies against oxidized cardiolipin are negatively associated with risk for development of CVD. Individuals (men and women) with low levels of anti-OxCL (below lowest quartile) had an almost 2-fold increased risk, independent of other risk factors. Further, high levels of IgM anti-OxCL were protective: values above 86^th^ percentile were associated with a more than 50% reduction of risk of CVD. The associations were especially striking for men; when analysed separately, the risk of development of MI was almost 2.5 in the lowest quartile and even though stroke cases were relatively few, it is interesting to note that the risk of stroke was 12-fold for men with anti-OxCL in the lowest quartile (though confidence intervals were high). In addition, men who within 5–7 years developed CVD had significantly lower median values at baseline than men who did not. There were also significant associations for women, where a two-fold risk for MI was noted below the highest quartile of anti-OxCL levels.

Further, we report that IgG anti-OxCL were not associated with CVD when divided in quartiles, however, very high antibody levels were indeed strong protection markers, with a decreased risk of CVD (OR of 0.23 above 95th percentile).

CL is abundant in the environment of mitochondrial oxidative burst, and is easily oxidized due to its unsaturated double bonds [16,17]. Further, it was demonstrated that cardiolipin is the only phospholipid in mitochondria undergoing early oxidation during apoptosis, by a cardiolipin-specific peroxidase activity of cardiolipin-bound cytochrome c [18,19]. Oxidized cardiolipin is needed for release of proapoptotic factors and thus may play a role in induction of apoptosis, eg through effects on release of cytochrome c, since cytochrome c anchorage in mitochondria depends on CL, and CL loss and/or oxidation can induce detachment and release, leading to apoptosis [18-20]. Though the OxCL studied herein is extensively oxidized it is thus still possible that similar forms of OxCL are present in vivo. The non-mutually exclusive possibility that enzymes such as phospholipase A2 may play an important role in CL-modification (as in LDL-modification) is also interesting and deserves further studies.

Our findings are comparable with our recent observations on another phospholipid-related epitope, phosphorylcholine (PC). We have reported that antibodies to PC (anti-PC) are negatively associated with CVD and atherosclerosis, with the most striking observations at low levels, which are associated to increased risk of CVD [5-8,21] while high levels are associated with a favourable development with less atherosclerosis [22]. It should be noted that PC is not present in CL (and thus not in OxCL) and further, cross reactivity between these antigens was relatively low (unpublished observation). In contrast to anti-PC, anti-OxCL was a protective factor for CVD at high levels, since high levels were associated with a 50% decreased risk.

In an interesting report it was demonstrated that CL is quickly oxidized when coated on ELISA-plates for determination of anti-CL and many anti-CL may recognize this variant of OxCL which could contribute to the antiphospholipid antibody syndrome [17]. Further, this form of CL, oxidized by exposure to air, had ß2GPI as a co-factor [23]. Interestingly, ß2GPI does not appear to play a role in antibody binding to OxCL. However, we cannot exclude that other protein-OxCL complexes could play a role, but if so, the antigen is not recognized by atherotrhombogenic antibodies.

The nature of the antigen recognized by anti-CL is still debated, almost three decades after their discovery [24], but there are many studies indicating that the plasma co-factor ß2GPI increases the binding to CL and plays a role in the antigenicity and also thrombogenicity.

The oxCL we used did not show a high cross-reactivity with CL as antigen. The commercial kit used herein for determination of antibodies against CL is developed to detect native CL. In contrast to IgM anti-OxCL, IgM anti-CL was not significantly associated with CVD either negatively or positively in the whole study group, though among men, median level of anti-CL IgM was lower among cases. However, there were trendwise associations between high IgM anti-CL-levels and increased risk of CVD among men and almost threefold but non-significant risks for women, when the highest percentiles were studied. Further, IgG anti-CL (which in general is believed to be more prothrombotic than IgM [11]), were not associated with CVD, not even trendwise. Whether some of the anti-OxCL binding to OxCL are present in immune complexes is an interesting possibility, which is beyond the scope of the present paper, which focused on the totality of oxCL binding antibodies.

The role of anti-CL in the general population is not clear, though such antibodies may play a role in subgroups as in survivors of MI (below 45 years of age) [25]. In one study on recurrent coronary events among patients with a history of MI, high IgG anti-CL, but also, interestingly, low IgM anti-CL were associated with increased risk [26]. In our study of 60-year olds with no history of MI or CVD, we did not determine such associations and determinations also of anti-OxCL in patients with a history of MI and/or CVD are clearly of interest. However, it appears likely that anti-CL mainly plays a role as a risk factor for CVD (including both arterial and venous thrombosis) in systemic rheumatic diseases like SLE where such antibodies are strong risk factors for CVD [11,27], and possibly among individuals with a history of CVD.

To identify mechanisms which could explain the protective role of anti-OxCL, we determined interactions with oxLDL, a major factor in atherosclerosis. OxLDL could inhibit antibody binding to oxCL but not to CL, which indicates cross reactivity. In an interesting previous report, it was demonstrated that aOxLDL cross react with anti-CL [28]. The most likely explanation to this difference is that CL is to some extend oxidized during the incubation used in that publication [17]. The finding that oxLDL cross reacts with oxCL could have several implications. Since CL is present in LDL [29], our finding most likely indicates that oxCL is a component of oxLDL. We recently demonstrated that oxCL but not CL is pro-inflammatory (unpublished observation) and one possibility is therefore that anti-OxCL is anti-inflammatory.

We demonstrated herein is that oxCL but not CL inhibits uptake of oxLDL in macrophages. OxLDL is taken up through specific scavenger receptors, which are not down-regulated when exposed to increasing amounts of oxLDL (as opposed to the uptake of LDL). Inhibition of the scavenger function is generally believed to be atheroprotective, preventing foam cell formation in the vascular wall which is a key process in development of atherosclerosis. In line with this, mice defective in scavenger receptor function develop less atherosclerosis [30]. Binding and uptake of oxLDL through oxCL could thus promote atherogenesis.

It should be noted, however, that recent research indicates that different scavenger receptors may play different roles and the role of scavenger receptors may vary depending on disease stage and type [31].

It has previously been demonstrated that murine monoclonal IgM recognizing forms of ox-CL can discriminate apoptotic cells from healthy cells [32]. Further, natural IgM binding to apoptotic cells increase apoptotic cell phagocytosis and also have direct anti-inflammatory properties [33]. It is possible that natural IgM including anti-PC and aOxCL could counter atherosclerosis development by increasing phagocytosis of dead and dying cells in the lesions.

It is interesting to note that the associations here were independent on traditional risk factors, and also hsCRP. Further, presence of hypertension in combination with low anti-OxCL increased the risk.

## Conclusions

Low anti-OxCL could be developed into a method to identify individuals at risk of CVD where intensified established treatment could be of importance, a possibility that further studies are needed to confirm. In the future, immunomodulation to raise levels of anti-OxCL through active or passive immunisation could be of interest.

## Competing interests

JF is named as inventor on patent applications relating to phospholipids and antibodies to them.

## Authors’ contributions

JS carried out and helped planning the experiments and revised the manuscript, XH participated in carrying out experiments, revised the manuscript, MV had main responsibility for statistics which was done together with UdF and JF, KL, BG, MLH and UdF revised the manuscript, UdF participated in designing the study together with JF, JF drafted the manuscript, planned experiments and design of study. All authors approved of the final version of the manuscript.

## Pre-publication history

The pre-publication history for this paper can be accessed here:

http://www.biomedcentral.com/1471-2261/13/1/prepub
